# Impact of metabolic dysfunction-associated steatotic liver disease on hepatocellular carcinoma risk in autoimmune hepatitis

**DOI:** 10.1371/journal.pone.0325066

**Published:** 2025-07-22

**Authors:** Jihye Lim, Ye-Jee Kim, Sehee Kim, Ju Hyun Shim

**Affiliations:** 1 Division of Gastroenterology and Hepatology, Department of Internal Medicine, Yeouido St. Mary’s Hospital, College of Medicine, The Catholic University of Korea, Seoul, Republic of Korea; 2 Department of Clinical Epidemiology and Biostatistics, Asan Medical Center, University of Ulsan College of Medicine, Seoul, Republic of Korea; 3 Department of Gastroenterology, Liver Center, Asan Medical Center, University of Ulsan College of Medicine, Seoul, Republic of Korea; Al-Azhar University, EGYPT

## Abstract

Few large-scale studies have investigated factors associated with the development of hepatocellular carcinoma (HCC) in patients with autoimmune hepatitis (AIH). This study aimed to determine the risk of HCC in AIH patients and associated risk factors, focusing on metabolic dysfunction-associated steatotic liver disease (MASLD). We analyzed the claims data from the Korean National Health Insurance Service from 2007 to 2020. The study included 7,382 patients with AIH and a control group of 58,538 age- and sex-matched individuals, at a ratio of 1:8. We compared the incidence rates of HCC between these groups and investigated the risk factors of HCC. During a median follow-up of 5.9 years, 160 AIH patients were diagnosed with HCC, resulting in an incidence rate of 3.60 per 1,000 person-years. The matched controls exhibited an incidence rate of 0.48 per 1,000 person-years. After adjustment, AIH patients had a 4.85-fold heightened risk of HCC compared to the control group. Within the AIH cohort, the presence of coexisting MASLD further elevated the risk of HCC, along with other factors such as older age, male sex, and decompensated liver cirrhosis, as observed in a two-year landmark analysis. The presence of concurrent extrahepatic autoimmune diseases did not affect the prognosis, while glucocorticoid treatment was associated with a decreased risk of HCC. Patients with AIH had an increased risk of HCC compared to matched controls, particularly those with coexisting MASLD. In addition to appropriate medical treatment, proactive interventions and lifestyle modifications for concurrent MASLD are recommended for these patients.

## Introduction

Autoimmune hepatitis (AIH) is a rare inflammatory liver disease of unknown etiology. [1–3] Over time, the prevalence of AIH has increased, with recent estimates reaching 17.4 cases per 100,000 individuals. [4] AIH manifests in genetically susceptible individuals due to a breakdown in self-tolerance to hepatocyte autoantigens, which is often triggered by environmental factors. The resulting immune response leads to progressive necroinflammation and fibrosis of the liver. [1] The clinical presentation of AIH can vary among individuals and may progress to cirrhosis and hepatic failure unless treated. [1–3,5]

The role of AIH as a risk factor for hepatocellular carcinoma (HCC) remains a topic of debate. Given the immunologic dysregulation and necroinflammation inherent to AIH—both of which are known to promote tumorigenesis—concerns have been raised regarding a potential link to HCC. [6] In fact, the estimated risk of HCC among AIH patients is less than 3%, which, while higher than that in the general population, is still lower than the risk associated with other hepatopathies. [7] Furthermore, the reported incidence of HCC in AIH patients varies significantly: a German study reported an incidence of 0.26 per 1,000 person-years, while an American study reported an incidence of 11.34 per 1,000 person-years. [8] Yet, the risk of HCC in patients with AIH within the Asian population remains largely unknown.

In recent years, metabolic dysfunction-associated steatotic liver disease (MASLD), a new term of nonalcoholic fatty liver disease, has emerged as a leading cause of HCC, exacerbated by increasingly sedentary lifestyles. The prevalence of MASLD in Korea is approximately 30% and is expected to rise. [9] Excessive fat accumulation in hepatocytes induces necroinflammation through mechanisms such as insulin resistance, oxidative stress, alterations in the immune system, genetic variations, and gut dysbiosis, which sequentially progress to hepatic steatosis, cirrhosis, and HCC. [10] Furthermore, excess adipose tissue contributes to the production of proinflammatory cytokines, inhibits apoptosis, and fosters a tumorigenic environment. [11] Notably, MASLD is now associated with over 10% of HCC cases, gradually surpassing chronic viral hepatitis in significance. [12] For patients with chronic viral liver diseases, the coexistence of MASLD independently increases the risk of developing HCC. [13]

We aimed to conduct a comprehensive assessment of HCC risk in patients with AIH. Our study compared the cumulative incidence of HCC in AIH patients to that in the general population, utilizing data from the Korea National Health Insurance Service (NHIS). We analyzed relevant risk factors in both the AIH cohort and the broader population, placing particular emphasis on the influence of MASLD on the occurrence of HCC.

## Materials and methods

### Study design and population

We conducted a nationwide population-based cohort study in the Republic of Korea. Our primary goal was to investigate the risk of HCC and its associated risk factors in patients diagnosed with AIH, and to compare this risk with that of a non-AIH reference population. The data for our analysis were sourced from the Korea NHIS. [14] The universal health coverage system in Korea, implemented in 1989, has consistently provided coverage for the entire population. Currently, the health insurance claims data encompass an impressive 1.3 trillion health information records dating back to 2002. This dataset offers a comprehensive overview, including demographics, socioeconomic status based on monthly household incomes, diagnoses (following the International Classification of Diseases, 10th Revision, ICD-10 guidelines), detailed treatment and prescription information, medical check-up outcomes, and behavioral and habitual data derived from questionnaires. It also includes registered data for cancer and rare incurable diseases (RIDs). [15]

Our study population was meticulously curated, consisting of adult patients diagnosed with AIH between 2007 and 2020, along with a matched cohort. AIH patients were identified using the ICD-10 code (K75.4) and the RID registration code (V121). To provide financial support for patients with RIDs, South Korea has implemented a unique case estimate system under which patients diagnosed and registered with RIDs benefit from a reduced co-payment rate of 10% for both outpatient and inpatient medical services. [14] The credibility of the RID system is bolstered by requiring physician-validated diagnoses based on biochemical, histological, and clinical evidence. Importantly, an AIH diagnosis relies on specific laboratory and histological findings, including elevated levels of aminotransferases and immunoglobulin G, the presence of characteristic autoantibodies, and defined histological abnormalities that exclude evidence of other liver diseases. These markers are crucial and align with the international diagnostic criteria for AIH. [1–3]

From our initial cohort of AIH patients, several exclusions were made. First, we removed 1,924 patients who were diagnosed before 2007. We then excluded 20 patients due to incomplete medical records. Additionally, 1,332 patients diagnosed with other chronic liver diseases, such as primary biliary cholangitis and chronic hepatitis B, were excluded. Furthermore, 49 patients with a history of malignancy, 4 patients diagnosed with HIV infection, and 655 patients with previous organ transplantation histories were also excluded.

**Fig 1:**
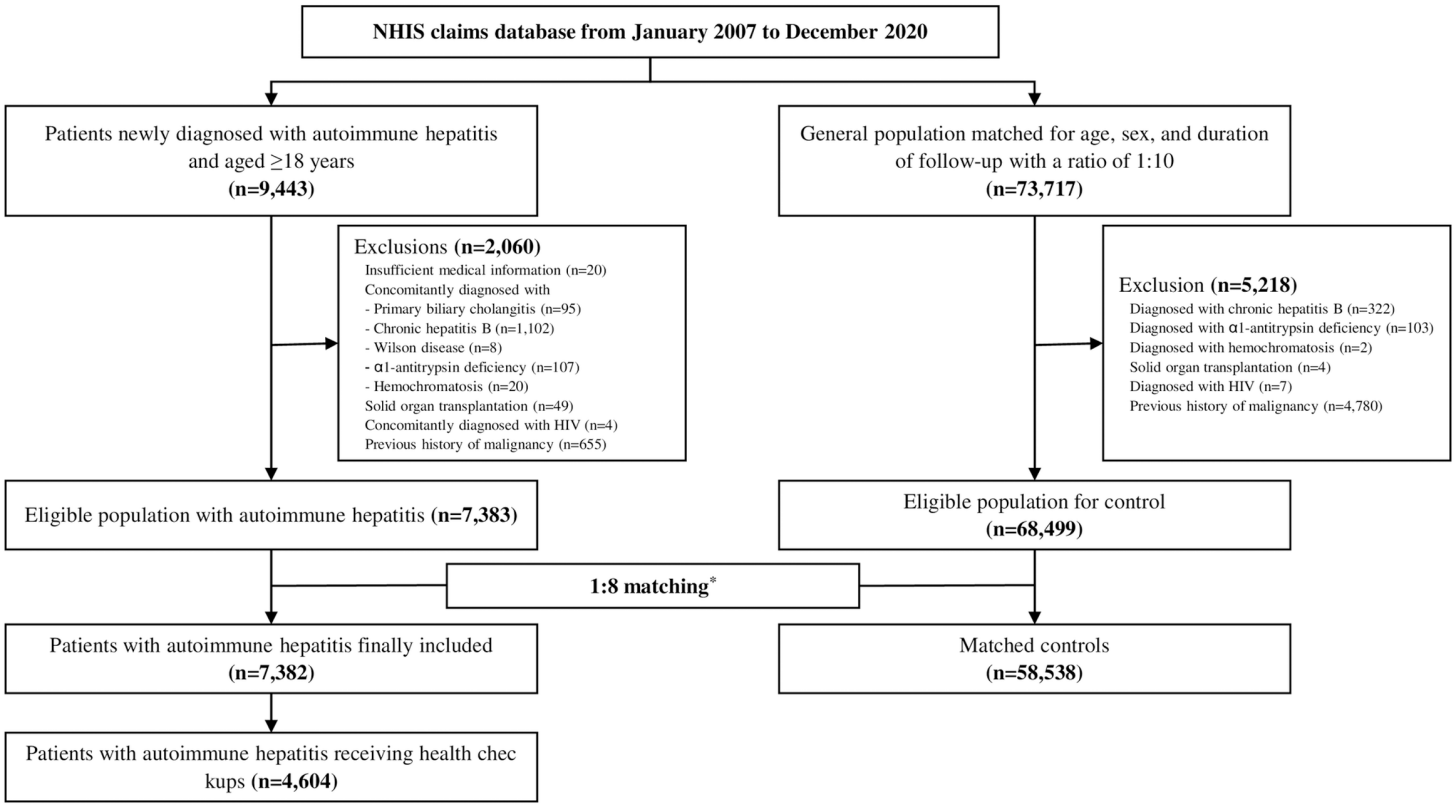
Study flow. Matched controls were randomly selected in a 1:8 ratio to pair with patients diagnosed with autoimmune hepatitis. The matching criteria included age (±2 years), sex, follow-up duration (0–2 years), and history of health check-ups. Abbreviations: HIV, human immunodeficiency virus; NHIS, National Health Insurance Service.

Finally, our study included 7,382 patients with AIH. We also selected 68,499 non-AIH individuals, matched for sex, age, and follow-up duration at a 1:8 ratio. The same exclusion criteria were applied to this non-AIH cohort (Fig 1). Our study was approved by the Institutional Review Board of the Catholic University of Korea (IRB No. SC23ZIDE0130). The data were accessed for research purposes from January to August 2024 in a closed network environment provided by the NHIS. Informed consent was not required, as we only utilized de-identified data.

### Data collection

We collected data on variables such as age, sex, socioeconomic status, comorbidities, drug prescriptions, and medical procedures from the NHIS claims data for this cohort. Comorbidities reported within one year prior to the diagnosis of AIH were assessed. Using this information, we quantified the total comorbidity burden using the Charlson Comorbidity Index (CCI). Liver cirrhosis was identified when the ICD-10 code K74 was referenced. Patients with cirrhosis who underwent treatment for ascites (via paracentesis or spironolactone) or experienced variceal bleeding (treated with endoscopy, terlipressin, or somatostatin) were classified as having decompensated cirrhosis.

The prescription of glucocorticoids (including medications such as prednisone, prednisolone, methylprednisolone, deflazacort, betamethasone, dexamethasone, and hydrocortisone) and other immunosuppressive agents (such as azathioprine, mercaptopurine, mycophenolate mofetil, tacrolimus, and cyclosporine) that lasted more than 180 days within the two years following the diagnosis of AIH was classified as medication use.

We obtained information regarding body measurements, blood pressure readings, blood tests, and lifestyle habits from the NHIS medical check-up data. The NHIS provides free health check-ups for eligible individuals. [14] Every Korean aged 20 and over is eligible for a general health check-up once every two years. Questionnaires are utilized to evaluate lifestyle habits, which include variables such as alcohol consumption, smoking, and exercise. Participation in these check-ups was recorded at 74.2% in 2021. [16]

We defined MASLD as hepatic steatosis characterized by a Fatty Liver Index (FLI) greater than 60, the presence of at least one of five cardiometabolic risk factors, and alcohol consumption of less than 420 mg/week for males and 350 mg/week for females. The FLI was calculated using the following equation: (e^0.953*loge (triglycerides) + 0.139*BMI + 0.718*loge (ggt) + 0.053*waist circumference – 15.745^)/(1 + e^0.953*loge (triglycerides) + 0.139*BMI + 0.718*loge (ggt) + 0.053*waist circumference – 15.745^) * 100. [17] Cardiometabolic risk factors were defined as follows: 1) body mass index (BMI) ≥23 kg/m^2^ or waist circumference >94 cm for males and >80 cm for females; 2) fasting serum glucose levels ≥100 mg/dL or a diagnosis of diabetes; 3) blood pressure ≥130/85 mmHg or a diagnosis of hypertension; 4) triglycerides ≥150 mg/dL or a diagnosis of dyslipidemia; 5) high-density lipoprotein (HDL) cholesterol ≤40 mg/dL for men and ≤50 mg/dL for women, or a diagnosis of dyslipidemia. [18] S1 Table provides the ICD-10 codes and operational definitions used in this study.

### Study outcomes

Our primary objective was to investigate the risk of HCC and its associated risk factors in patients diagnosed with AIH, and to compare this risk with that of a non-AIH reference population. The primary outcome was the development of HCC during the designated follow-up period. HCC was defined as a claim associated with the ICD-10 code C22.0, along with the presence of cancer-specific RID codes, namely V193, V194, and V027. [14] As a secondary outcome, we examined the rate of all-cause mortality. [15]

### Statistical analysis

The control group consisted of individuals without AIH in a 1:8 ratio with AIH patients, matched for age (±2 years), sex, health check-up, and follow-up duration (0–2 years) using the Greedy method to minimize potential immortal time bias. For baseline characteristics, categorical variables are presented as counts and percentages, while continuous variables are reported as mean ± standard deviation (SD) or as median with interquartile range.

The index date for the AIH group was the date of the first RID registration, which corresponded to the matched date for the control group. Follow-up for participants continued from the index date until either death, immigration, or the end of the study in December 2020.

We calculated the incidence rate of HCC per 1,000 person-years and estimated 95% confidence intervals (CIs) based on the Poisson distribution. Cumulative incidence curves were generated using the Kaplan-Meier method. To compare the risk of HCC between AIH patients and matched controls, we conducted a competing risk analysis, considering overall mortality as a competing risk. We employed Fine-Gray’s subdistribution hazard model with a robust variance estimator to account for matched pairs. The covariates adjusted for in the model included age, sex, socioeconomic status, presence of decompensated cirrhosis, extrahepatic autoimmune disease, healthcare check-ups, alcohol consumption, smoking, and MASLD. To identify risk factors for HCC in the AIH patient population, we performed a multivariable analysis including both variables with P values <0.05 in the univariate analysis and those considered clinically relevant. For the 2-year landmark analyses, we assessed subjects with a follow-up duration of at least 2 years to determine the long-term effects of MASLD and the correlation between cumulative use of glucocorticoids or immunosuppressive agents during the first 2 years after AIH diagnosis and the subsequent risk of developing HCC.

P-values less than 0.05 were considered to indicate statistical significance. All statistical analyses were performed using SAS Enterprise Guide Software Version 7.1 (SAS Institute, Inc., Cary, NC).

## Results

### Baseline characteristics of the entire cohort

Table 1 presents the baseline characteristics of the study population. The median duration of follow-up was 5.9 years (interquartile range [IQR], 3.3–9.6). Among the 65,920 study individuals, the average age was 56.4 ± 13.7 years and 85.1% were female.

**Table 1:** Baseline characteristics of patients diagnosed with autoimmune hepatitis (n = 7,382) and matched controls (n = 58,538).

	Overall	Autoimmune hepatitis	Matched control	P value
	(n = 65,920)	(n = 7,382)	(n = 58,538)	
Age years, mean (SD)	56.4 (13.7)	56.4 (13.8)	56.4 (13.7)	0.988
Female sex, n (%)	56,108 (85.1)	6,268 (84.9)	49,840 (85.1)	0.598
Diagnosis period, n (%)				0.756
2007–2010	15,125 (22.9)	1,717 (23.3)	13,408 (22.9)	
2011–2015	23,293 (35.3)	2,587 (35.0)	20,706 (35.4)	
2016–2019	27,502 (41.7)	3,078 (41.7)	24,424 (41.7)	
Socioeconomic status, n (%)				<0.001
National health insurance	61,417 (93.2)	6,877 (93.2)	54,540 (93.2)	
Household income ≥70%	25,434 (38.6)	3,126 (42.3)	22,308 (38.1)	
Household income 30–70%	21,263 (32.3)	2,299 (31.1)	18,964 (32.4)	
Household income <30%	14,720 (22.3)	1,452 (19.7)	13,268 (22.7)	
Medical aid	2,765 (4.2)	364 (4.9)	2,401 (4.1)	
Unknown	1,738 (2.6)	141 (1.9)	1,597 (2.7)	
Comorbidities, n (%)				
Hypertension	21,353 (32.4)	2,940 (39.8)	18,413 (31.5)	<0.001
Diabetes mellitus	13,084 (19.8)	2,603 (35.3)	10,481 (17.9)	<0.001
Dyslipidemia	22,893 (34.7)	4,902 (66.4)	17,991 (30.7)	<0.001
CCI score, median (IQR)	1.0 [0.0, 2.0]	3.0 [2.0, 4.0]	1.0 [0.0, 2.0]	<0.001
Decompensated cirrhosis, n (%)	724 (1.1)	640 (8.7)	84 (0.1)	<0.001
Extrahepatic autoimmune disease, n (%)	6,924 (10.5)	2,187 (29.6)	4,737 (8.1)	<0.001
*Available for health check-ups, n (%)*	*47,591 (72.2)*	*5,458 (73.9)*	*42,133 (72.0)*	*<0.001*
MASLD, n (%)				<0.001
With MASLD	4,167 (6.3)	1,010 (13.7)	3,157 (5.4)	
Without MASLD	36,915 (56.0)	3,594 (48.7)	33,321 (56.9)	
Unknown	24,838 (37.7)	2,778 (37.6)	22,060 (37.7)	
Fatty liver index, n (%)				<0.001
>60	4,271 (6.5)	1,021 (13.8)	3,250 (5.6)	
60≤	36,811 (55.8)	3,583 (48.5)	33,228 (56.8)	
Unknown	24,838 (37.7)	2,778 (37.6)	22,060 (37.7)	
Body mass index ≥23 kg/m^2^ or waist circumference ≥90 cm (for men) and ≥85 cm (for women), n (%)				<0.001
Yes	28,462 (43.2)	3,294 (44.6)	25,168 (43.0)	
No	19,125 (29.0)	2,163 (29.3)	16,962 (29.0)	
Unknown	18,333 (27.8)	1,925 (26.1)	16,408 (28.0)	
Fasting serum glucose ≥100 mg/dL or diabetes, n (%)				<0.001
Yes	22,195 (33.7)	3,244 (43.9)	18,951 (32.4)	
No	23,215 (35.2)	2,229 (30.2)	20,986 (35.9)	
Unknown	20,510 (31.1)	1,909 (25.9)	18,601 (31.8)	
Blood pressure ≥130/85 mm Hg or hypertension, n (%)				<0.001
Yes	26,340 (40.0)	3,325 (45.0)	23,015 (39.3)	
No	21,840 (33.1)	2,257 (30.6)	19,583 (33.5)	
Unknown	17,740 (26.9)	1,800 (24.4)	15,940 (27.2)	
Triglycerides ≥150 mg/dL or dyslipidemia, n (%)				<0.001
Yes	28,263 (42.9)	5,112 (69.2)	23,151 (39.5)	
No	19,028 (28.9)	1,062 (14.4)	17,966 (30.7)	
Unknown	18,629 (28.3)	1,208 (16.4)	17,421 (29.8)	
High-density lipoprotein cholesterol ≤40 mg/dL (for men) and ≤50 mg/dL (for women) or dyslipidemia, n (%)				<0.001
Yes	28,652 (43.5)	3,047 (41.3)	25,605 (43.7)	
No	12,430 (18.9)	1,557 (21.1)	10,873 (18.6)	
Unknown	24,838 (37.7)	2,778 (37.6)	22,060 (37.7)	
Alcohol consumption (gram/week, n [%])				
Non–drinker	34,123 (51.8)	4,388 (59.4)	29,735 (50.8)	<0.001
<210 (for males) or <140 (for females)	10,680 (16.2)	834 (11.3)	9,846 (16.8)	
210– < 420 (for males) or 140– < 350 (for females)	1,413 (2.1)	107 (1.4)	1,306 (2.2)	
≥420 (for males) or ≥350 (for females)	725 (1.1)	42 (0.6)	683 (1.2)	
Unknown	18,979 (28.8)	2,011 (27.2)	16,968 (29.0)	
Smoking, n (%)				<0.001
Never	40,503 (61.4)	4,658 (63.1)	35,845 (61.2)	
Past	2,761 (4.2)	358 (4.8)	2,403 (4.1)	
Current	3,754 (5.7)	366 (5.0)	3,388 (5.8)	
Unknown	18,902 (28.7)	2,000 (27.1)	16,902 (28.9)	

Abbreviations: CCI, Charlson Comorbidity Index; IQR, interquartile range; MASLD, metabolic dysfunction-associated steatotic liver disease; SD, standard deviation.

The AIH group exhibited a significantly higher prevalence of comorbidities than controls, including hypertension, diabetes mellitus, and dyslipidemia, as indicated by a higher CCI score (3.0 vs. 1.0; all P < 0.001). The incidence of decompensated cirrhosis was significantly higher in the AIH group than in the matched control group (8.7% vs. 0.1%; P < 0.001). Furthermore, extrahepatic autoimmune diseases were more common in the AIH group as well (29.6% vs. 8.1%; P < 0.001) (S2 Table). The AIH group also had a higher prevalence of MASLD compared to matched controls (13.7% vs. 5.4%, P* *< 0.001).

### HCC incidence and mortality in AIH vs. matched control

**Fig 2:**
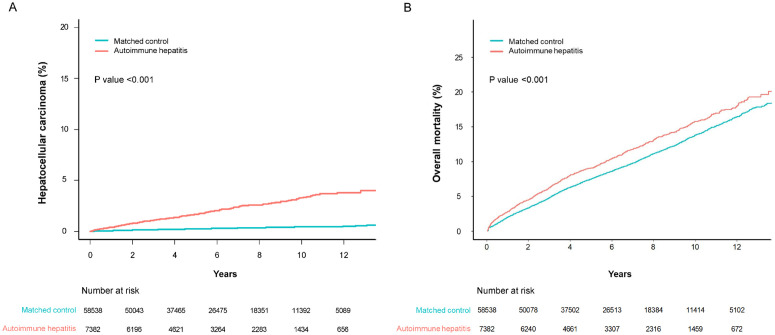
Event frequencies and Kaplan-Meier estimates for (A) hepatocellular carcinoma and (B) overall mortality in patients with autoimmune hepatitis and matched controls.

During the study period, 160 out of 7,382 AIH patients were diagnosed with HCC, resulting in an incidence rate of 3.60 cases per 1,000 person-years (95% CI: 3.06–4.20). In contrast, 170 out of 58,538 individuals in the control group were diagnosed with HCC, resulting in an incidence rate of 0.48 cases per 1,000 person-years (95% CI: 0.41–0.56). The risk of developing HCC in AIH patients was significantly higher than that in matched controls at the following time points: 1 year (0.4% vs. 0.1%), 5 years (1.7% vs. 0.2%), and 10 years (3.3% vs. 0.4%). These risks correspond to a crude subhazard ratio (SHR) of 7.40 (95% CI: 5.97–9.17; Fig 2A and Table 2). After adjusting, AIH patients exhibited a 4.85-fold elevated risk of HCC compared to the controls (95% CI: 3.64–6.47; Table 2).

**Table 2:** Risk factors for hepatocellular carcinoma in patients with autoimmune hepatitis (n = 7,382) and matched controls (n = 58,538).

	Univariate analysis		Multivariable analysis	
	SHR (95% CI)	P value^*^	SHR (95% CI)	P value^*^
Autoimmune hepatitis	7.40 (5.97–9.17)	<0.001	4.85 (3.64–6.47)	<0.001
Age	1.05 (1.04–1.06)	<0.001	1.04 (1.03–1.05)	<0.001
Sex				
Female	Ref	–	Ref	–
Male	2.06 (1.60–2.66)	<0.001	2.09 (1.62–2.70)	<0.001
Socioeconomic status				
Household income ≥70%	Ref	–	Ref	–
Household income 30–70%	0.92 (0.71–1.18)	0.51	1.12 (0.87–1.45)	0.389
Household income <30%	0.76 (0.56–1.02)	0.069	0.99 (0.73–1.34)	0.935
Medical aid	1.50 (0.97–2.34)	0.071	1.22 (0.78–1.93)	0.383
Unknown	0.86 (0.42–1.76)	0.687	1.07 (0.52–2.21)	0.848
Decompensated cirrhosis	22.75 (17.27–29.97)	<0.001	4.94 (3.39–7.22)	<0.001
Extrahepatic autoimmune disease	1.96 (1.48–2.60)	<0.001	1.08 (0.80–1.44)	0.623
Health check-up	0.92 (0.73–1.16)	0.481		
MASLD				
Without MASLD	Ref	–	Ref	–
With MASLD	3.61 (2.55–5.10)	<0.001	1.88 (1.30–2.71)	0.001
Unknown	1.48 (1.15–1.91)	0.002	1.41 (1.09–1.83)	0.010
Alcohol quantity (gram/week)				
Non–drinker	Ref	–		
<210 (for males) or <140 (for females)	0.44 (0.28–0.69)	<0.001		
210– < 420 (for males) or 140– < 350 (for females)	1.57 (0.86–2.88)	0.146		
≥420 (for males) or ≥350 (for females)	0.18 (0.03–1.32)	0.092		
Unknown	1.05 (0.83–1.34)	0.665		
Smoking				
Never	Ref	–		
Past	1.81 (1.11–2.93)	0.017		
Current	2.19 (1.51–3.18)	<0.001		
Unknown	1.33 (1.04–1.69)	0.021		

Abbreviation: CI, confidence interval; MASLD, metabolic dysfunction-associated steatotic liver disease; SHR, subhazard ratio.

* P-values were calculated using Fine-Gray’s subdistribution hazard models with a robust variance estimator to account for matched pairs.

Several factors were identified as contributing to the increased risk of HCC, including older age (adjusted SHR [95% CI]: 1.04 [1.03–1.05]), male sex (2.09 [1.62–2.70]), presence of decompensated cirrhosis (4.94 [3.39–7.22]), and the presence of MASLD (1.88 [1.30–2.71]).

The risk of death was significantly higher in AIH patients than in matched controls at the following time points: 1 year (2.8% vs. 2.0%), 5 years (9.1% vs. 7.5%), 10 years (15.7% vs. 13.8%) (all P < 0.001; Fig 2B).

### Two–year landmark analysis for risk factors associated with HCC in AIH patients

Recorded data regarding MASLD and lifestyle habits were available for 4,604 (62.4%) of the AIH patients. Within this subset, 3,805 patients with AIH (82.6%) who were followed for more than two years were included in the landmark analysis. S3 Table presents the baseline characteristics of 2,979 patients without MASLD (78.3%) and 826 patients with MASLD (21.7%). Patients with MASLD were older and predominantly male. The rates of comorbidities and decompensated cirrhosis were higher in patients with MASLD than in those without MASLD. Patients without MASLD had a greater incidence of extrahepatic immune diseases than those with MASLD. Medication use was similar between the two groups. Most patients with AIH (99.4%) consumed alcohol less than 420 mg/week for males or 350 mg/week for females.

**Fig 3:**
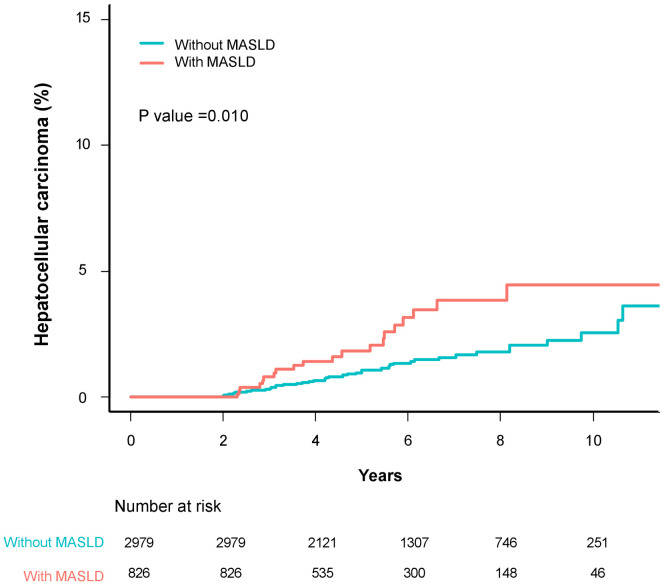
Two-year landmark analysis of event frequencies and Kaplan-Meier estimates for hepatocellular carcinoma in patients with autoimmune hepatitis stratified by the presence of MASLD. Abbreviations: MASLD, metabolic dysfunction-associated steatotic liver disease.

Table 3 lists the factors associated with the development of HCC in AIH patients. Over a median follow-up duration of 5.8 ± 2.6 years, 61 patients (20 with MASLD, 41 without MASLD) were diagnosed with HCC. After adjustment, several factors emerged as significant contributors to HCC development in a two-year landmark analysis, including age (adjusted SHR [95% CI]: 1.07 [1.04–1.09]), male sex (2.47 [1.55–5.35]), and decompensated cirrhosis (2.60 [1.24–5.45]). Additionally, the presence of MASLD showed marginal significance for HCC occurrence (1.74 [1.00–3.04]). Regarding the cumulative incidence of HCC among AIH patients with MASLD, those with MASLD had a higher risk of developing HCC: 1.8% vs. 1.1% at 5 years (P = 0.090) and 4.5% vs. 2.5% at 10 years (P = 0.007) (Fig 3). The use of glucocorticoids (adjusted SHR [95% CI]: 0.54 [0.30–0.97]) was associated with a decreased risk of HCC, while the use of immunosuppressive agents had no significant impact on HCC risk (1.76 [0.98–3.19]). Notably, extrahepatic autoimmune diseases did not show a significant correlation with the risk of HCC (1.24 [0.71–2.18]).

**Table 3:** Two-year landmark analysis of risk factors associated with the development of hepatocellular carcinoma in patients with autoimmune hepatitis (n = 3,805).

	Model^a^		Model^b^		Model^c^		Model^d^		Model^e^	
	SHR (95% CI)	P value^*^	SHR (95% CI)	P value^*^	SHR (95% CI)	P value^*^	SHR (95% CI)	P value^*^	SHR (95% CI)	P value^*^
MASLD										
Without MASLD	Ref	–	Ref	–	Ref	–	Ref	–	Ref	–
With MASLD	2.01 (1.18-3.42)	0.010	1.74 (1.00-3.02)	0.050	1.79 (1.03-3.10)	0.040	1.80 (1.03-3.12)	0.038	1.74 (1.00-3.04)	0.050
Age			1.07 (1.05-1.09)	<0.001	1.07 (1.05-1.10)	<0.001	1.07 (1.05-1.10)	<0.001	1.07 (1.04-1.09)	<0.001
Sex										
Female			Ref	–	Ref	–	Ref	–	Ref	–
Male			2.49 (1.40-4.40)	0.002	2.49 (1.15-5.41)	0.021	2.52 (1.17-5.44)	0.019	2.47 (1.15-5.35)	0.021
Socioeconomic status										
Household income ≥30%					Ref	–	Ref	–	Ref	–
Household income <30%, Medical aid, or unknown					0.90 (0.49-1.68)	0.744	0.90 (0.49-1.68)	0.744	0.92 (0.49-1.72)	0.784
Alcohol (gram/week)										
<420 (for males) or <350 (for females)					Ref	–	Ref	–	Ref	–
≥420 (for males) or ≥350 (for females) or unknown					4.16 (0.52-33.26)	0.179	4.04 (0.49-33.19)	0.194	4.10 (0.49-34.67)	0.195
Smoking										
Never					Ref	–	Ref	–	Ref	–
Ever- or unknown					1.01 (0.44-2.30)	0.991	1.00 (0.44-2.29)	0.997	0.97 (0.42-2.21)	0.934
Extrahepatic autoimmune disease							1.15 (0.66-2.00)	0.622	1.24 (0.71-2.18)	0.458
Decompensated cirrhosis									2.60 (1.24-5.45)	0.011
Medication use^†^										
Glucocorticoid									0.54 (0.30-0.97)	0.038
Immunosuppressive agents^‡^									1.76 (0.98-3.19)	0.060

Abbreviation: CI, confidence interval; CI, confidence interval; MASLD, metabolic dysfunction-associated steatotic liver disease; SHR, subhazard ratio.

^a^Crude analysis.

^b^Adjusted for MASLD, age, and sex.

^c^Adjusted for MASLD, age, sex, socioeconomic status, alcohol, and smoking.

^d^Adjusted for MASLD, age, sex, socioeconomic status, alcohol, smoking, and extrahepatic autoimmune disease.

^e^Adjusted for MASLD, age, sex, socioeconomic status, alcohol, smoking, extrahepatic autoimmune disease, decompensated liver cirrhosis, and medication use.

* P-values were calculated using Fine-Gray’s subdistribution hazard models with a robust variance estimator to account for matched pairs.

† Medication use was defined as the prescription of medications for more than 180 days within the two years following the diagnosis of autoimmune hepatitis.

‡ Immunosuppressive agents included azathioprine, mercaptopurine, mycophenolate mofetil, tacrolimus, and cyclosporine, all of which were prescribed after the diagnosis of autoimmune hepatitis.

## Discussion

In this nationwide population-based cohort study in Korea, we found that patients with AIH exhibited an elevated risk of HCC and mortality compared to age- and sex-matched controls. This increased risk persisted regardless of the severity of MASLD. Key determinants contributing to the heightened risk of HCC in AIH patients included the concurrent presence of MASLD, advanced age, male sex, and decompensated cirrhosis. Glucocorticoid therapy was associated with a reduced risk of HCC in this population, while the presence of extrahepatic autoimmune diseases was not significantly associated with the risk of HCC.

An association between AIH and the risk of HCC has long been suspected; however, definitive evidence has remained elusive. A meta-analysis by Tarao et al. indicated that the annual incidence of HCC in patients with AIH was 0.19% in non-cirrhotic individuals and 0.53% in those with cirrhosis. These rates are substantially lower than those observed in other chronic liver diseases, such as chronic hepatitis B and C, which have respective rates of 0.37% and 0.68% in non-cirrhotic patients, and 3.23% and 4.81% in cirrhotic patients. [19] In our comprehensive cohort, we identified a clear association between AIH and an increased risk of HCC. Specifically, the incidence rate of HCC was 3.6 per 1,000 person-years among AIH patients, representing a 4.85-fold increased risk compared to the matched control group. This finding is consistent with previous meta-analyses by Yan et al. and European studies. [20–23] However, our data from Asia revealed a 10-year HCC risk that exceeds those reported in European studies, with rates of 3.3% for AIH patients and 0.4% for matched controls. A 2020 report on the global burden of primary liver cancer, which forecasts trends up to 2040, emphasized that the anticipated incidence of primary liver cancer in Asia surpasses that in Europe, with age-standardized rates per 100,000 individuals of 13.7 compared to 5.0–6.7, respectively. [24] This broader global context underscores the significance and credibility of our findings.

In our results, MASLD was associated with an elevated risk of HCC in patients with AIH. In the multivariable analysis, this association demonstrated marginal statistical significance, likely due to the small number of HCC events in our landmark study. Notably, Western data highlighting the impact of obesity on HCC development in AIH patients supports the causal relationships between MASLD—a hallmark feature of obese individuals—and the occurrence of HCC. [23] Furthermore, MASLD was directly correlated with increased mortality in AIH patients. An experimental study utilizing the CYP2D6 mouse model, which replicates several characteristic features of human AIH, such as interface hepatitis, hepatic fibrosis, and the formation of AIH-specific autoantibodies and CYP2D6-specific T cells, revealed that MASLD exacerbates AIH. [25] Mouse models of MASLD exhibited heightened liver injury characteristics, including increased cellular infiltration, pronounced hepatic fibrosis, and a surge in liver autoantigen-specific T cells. This intensified pro-inflammatory pathway may be crucial in driving the observed adverse prognosis. In fact, a multinational cohort study reported that the presence of concomitant MASLD—identified in 22.8% of 640 AIH patients—was associated with an increased risk of cirrhosis. [26] Hepatic steatosis, a feature of MASLD, has been identified as a strong prognostic factor in various chronic liver disorders. A study by Kim et al. emphasized that concurrent MASLD is an independent risk factor for both HCC and mortality in patients with chronic viral hepatitis. [13] Considering the association between MASLD and HCC development in our AIH cohort and the rising global prevalence of MASLD, [9] there is a critical need for proactive lifestyle interventions. Lifestyle modifications aimed at addressing obesity, insulin resistance, and dyslipidemia may help slow the progression of fibrosis and decrease the risk of HCC. Adopting a Mediterranean diet, participating in customized physical activity, and minimizing sedentary behavior are recommended strategies for achieving weight loss and managing MASLD. [27] In our study, the incidence of HCC in patients with AIH was 0.36%, and 0.55% in those with concurrent MASLD. Considering that the cost-effective surveillance threshold for HCC is ≥ 1.0% per year in patients with cirrhosis, [28] further studies are necessary to establish HCC surveillance guidelines for patients with AIH, particularly those with concurrent MASLD.

Previous investigations into the risk factors for HCC in AIH patients have often yielded inconclusive results. In particular, there is significant concern regarding the potential effects of various treatments, especially glucocorticoids and immunosuppressive agents. [1–3,23] While most AIH patients are thought to require medication for two years or more, [1–3] prolonged immunosuppression may create a pro-carcinogenic environment by inhibiting immune-mediated tumor surveillance, impairing DNA repair mechanisms, and limiting cancer cell apoptosis. [6] A Danish study reported that the risks of HCC associated with the drugs prednisolone and azathioprine were 0.98 (95% CI, 0.77–1.6) and 1.25 (95% CI, 0.90–1.73), respectively, both of which were inconclusive. [22] Another analysis of 227 AIH patients in the West indicated that more than three years of immunosuppressive treatment increased the HCC risk by a factor of 7.3. [29] In our research, the use of glucocorticoids was associated with a reduction in HCC risk of approximately 0.54-fold. Regarding the impact of specific responses to glucocorticoid-based therapy on HCC development, additional data, that take into account AIH disease activity and liver fibrosis status are necessary to confirm this issue in AIH patients. [23]

Glucocorticoids possess an inherent ability to reduce inflammation and mitigate overactive immune responses, thereby creating an opportunity for liver cell repair. [30] Glucocorticoids activate hepatic stellate cells and immune cells, suppress fibrotic gene expression, decrease extracellular matrix deposition, and inhibit pro-inflammatory signaling. By mitigating chronic necroinflammation and fibrosis, glucocorticoids may ultimately reduce the long-term risk of HCC. [31] Consistent with this, a longitudinal cohort study conducted in the UK demonstrated that glucocorticoid treatment significantly decreased both mortality rates and the necessity for liver transplantation. [32] Further studies incorporating cumulative dosage or duration of glucocorticoid exposure may help elucidate the effectiveness and safety of glucocorticoids. In contrast, immunosuppressive agents are typically reserved as secondary treatment options for patients who exhibit an inadequate response to, or intolerance of, standard treatments, including glucocorticoids. [1–3] However, the current literature regarding their effectiveness and safety remains inconclusive due to a lack of comprehensive studies. [33] This highlights the urgent need for further research to clarify the therapeutic impact of immunosuppressive agents and their potential side effects.

The coexistence of extrahepatic autoimmune diseases alongside AIH is not uncommon. [1–3] However, the clinical implications of this coexistence remain a topic of debate. A study conducted in Denmark involving 2,479 AIH patients found that the presence of extrahepatic autoimmune diseases resulted in a 1.30-fold increase in mortality over an 8.4-year follow-up period. [34] The increased mortality among Danish patients with AIH was attributed to a combination of symptomatic challenges and complications related to treatment. In contrast, a UK-based study involving 457 patients with AIH, of whom 194 (42.4%) had concurrent extrahepatic autoimmune diseases, did not find any significant impact of these diseases on the incidence of HCC or mortality rates. [35] Additionally, a noteworthy study from Sweden involving 5,268 patients with AIH revealed that 751 (14.3%) had extrahepatic autoimmune diseases, with 13 patients diagnosed with HCC. In the matched general population, the prevalence of extrahepatic autoimmune diseases was only 1.4%, and no HCC cases were reported. [21] Our findings diverge from some previous studies, as we observed that the presence of extrahepatic manifestations was not associated with a higher risk of HCC.

Our study has several limitations. First, due to the inherent limitations of claims data, we conducted an operational assessment of various liver diseases using ICD-10 and RID codes, which may have led to underdiagnosis or misclassification of these conditions. We also identified steatotic liver diseases, defined as an FLI greater than 60, since direct imaging methods such as ultrasonography or CT scans were unavailable; this approach may have introduced an ascertainment bias. Nonetheless, it is important to note that the FLI has consistently demonstrated effectiveness in predicting fatty liver disease across both Western and Asian populations. [36–38] In a Korean population, the FLI demonstrated an area under the receiver operating characteristic curve (AUROC) of 0.785 for detecting hepatic steatosis. [38] In a Japanese population, the AUROC of the FLI was 0.844, with a positive predictive value of 86.1% and a specificity of 64.8%. [39] Further validation of the association between FLI and MASLD warrants investigation. The FLI includes gamma-glutamyl transpeptidase as one of its variables; however, elevation of gamma-glutamyl transpeptidase is generally not significant in patients with AIH, [40] and its levels are not affected by the presence of cirrhosis. [41] Secondly, our database lacked specific laboratory and histological findings related to AIH disease activity. Despite this limitation, we were able to identify patients exhibiting decompensated cirrhosis, even though information on compensated cirrhosis and advanced fibrosis—a late-stage manifestation of AIH known to increase the risk of HCC and mortality—was not available; [42] as expected, we found that the presence of decompensated cirrhosis was associated with an elevated risk of HCC. Lastly, as our study was based on claims data, the diagnosis of AIH may have varied depending on the timing and the guidelines employed; additionally, the tumor stage of HCC at the time of diagnosis was not available. [1–3,23,43]

In conclusion, we found that Asian patients with AIH had a heightened risk of HCC. In the two-year landmark analysis of the AIH cohort, the presence of concurrent MASLD was associated with an increased risk of HCC. Furthermore, treatment with glucocorticoids appeared to play a significant role in preventing HCC. Based on these findings, we recommend that AIH patients, particularly those with coexisting MASLD, undergo rigorous monitoring, make proactive lifestyle modifications, and receive timely standard medical therapy for AIH *per se*.

## Supporting information

S1 Table Definitions of comorbidities used in this study. (DOCX)

S2 Table Distribution of extrahepatic autoimmune diseases in autoimmune hepatitis patients (n = 7,382) and matched cohort (n = 58,538). (DOCX)

S3 Table Baseline characteristics of patients with autoimmune hepatitis (n = 3805) stratified by the presence of MASLD in the two-year landmark analysis. (DOCX)
