# Supplementary material for: Impact of metabolic dysfunction-associated steatotic liver disease on hepatocellular carcinoma risk in autoimmune hepatitis
Source: PLoS One. 2025 Jul 22;20(7):e0325066. doi: 10.1371/journal.pone.0325066 (PMC12282895; doi:10.1371/journal.pone.0325066)
Supplement: S1 Table — (DOCX) [file pone.0325066.s001.docx]

**Supporting information**

**S1 Table. Definitions of comorbidities used in this study**

| **Disease** | **ICD-10 code** | **RID code** | **Procedure code** | **Drug code** |
| --- | --- | --- | --- | --- |
| Alpha-1 antitrypsin deficiency | E880 |  |  |  |
| Autoimmune hepatitis | K754 | V175 |  |  |
| Chronic hepatitis B | B18.0, B18.1 |  |  |  |
| Decompensated cirrhosis | M74 with management for varix or ascites |  | Endoscopic variceal bleeding control: Q7631, Q7633 | Terlipressin 236001BIJ, 236003BIJ, 236030BIJ |
|  |  |  |  | Somatostatin 230201BIJ, 230203BIJ, 230202BIJ |
|  |  |  | Paracentesis: C8050 | Spironolactone 231101ATB, 231102ATB |
| *Diabetes mellitus* | E10, E11, E12, E13, E14 |  |  |  |
| Dyslipidemia | E78 |  |  |  |
| Hepatocellular carcinoma | C220 | V193, V194, V027 |  |  |
| Hereditary haemochromatosis | E83.1 |  |  |  |
| Human Immunodeficiency Virus | B20.0, B20.2, B20.4, B20.6, B21.0, B21.1, B21.2, B22.0, B22.2, B24 |  |  |  |
| Hypertension | I10, I11, I12, I13, I14, I15 |  |  |  |
| Liver transplantation | Z94,4 T86.4 |  |  |  |
| Malignancy | C00–C97 | V193, V194, V027 |  |  |
| Primary biliary cholangitis | K74.3 | V174 |  |  |
| Primary sclerosing cholangitis | K83.0 | V262 |  |  |
| Rheumatoid arthritis | M05, M06 |  |  |  |
| Solid organ transplantation | Z94, T86 |  |  |  |
| Wilson disease | E83.0 | V119 |  |  |
| Extrahepatic autoimmune disease |  |  |  |  |
| Diseases of the blood and blood-forming organs |  |  |  |  |
| Vitamin B12 deficiency anemia due to intrinsic factor deficiency | D51.0 |  |  |  |
| Autoimmune hemolytic anemia | D59.0, D59.1 |  |  |  |
| Allergic purpura | D69.0 |  |  |  |
| Idiopathic thrombocytopenic purpura | D69.3 |  |  |  |
| Certain disorders involving the immune mechanism |  |  |  |  |
| Sarcoidosis | D86, G53.2, M63.3 |  |  |  |
| Autoimmune disease not elsewhere classified | D89.8, D89.9 |  |  |  |
| Endocrine disease |  |  |  |  |
| Thyrotoxicosis with diffuse goiter | E05.0 |  |  |  |
| Autoimmune thyroiditis | E06.3 |  |  |  |
| Type 1 diabetes mellitus | E10 |  |  |  |
| Primary adrenocortical insufficiency (Addison's disease) | E27.1 | V116 |  |  |
| Diseases of the nervous system |  |  |  |  |
| Multiple sclerosis | G35 |  |  |  |
| Guillain–Barre syndrome | G61.0 |  |  |  |
| Myasthenia gravis | G70.0 |  |  |  |
| Diseases of the eye |  |  |  |  |
| Iridocyclitis | H20 |  |  |  |
| Other endophthalmitis | H44.1 |  |  |  |
| Diseases of the circulatory system |  |  |  |  |
| Rheumatic fever | I00, I01 |  |  |  |
| Raynaud’s syndrome | I73.0 |  |  |  |
| Thromboangiitis obliterans | I73.1 |  |  |  |
| Diseases of the digestive system |  |  |  |  |
| Crohn's disease | K508, K501 K500 | V130 |  |  |
| Ulcerative colitis | K51 |  |  |  |
| Celiac disease | K90.0 |  |  |  |
| Diseases of the skin and subcutaneous tissue |  |  |  |  |
| Pemphigus | L10 |  |  |  |
| Pemphigoid | L12 |  |  |  |
| Dermatitis herpetiformis | L13.0 |  |  |  |
| Psoriasis | L40 |  |  |  |
| Erythema nodosum | L52 |  |  |  |
| Alopecia areata | L63 |  |  |  |
| Vitiligo | L80 |  |  |  |
| Lupus erythematosus | L93 |  |  |  |
| Other localized connective tissue disorders | L94.0, L94.1, L94.3 |  |  |  |
| Diseases of the musculoskeletal system and connective tissue |  |  |  |  |
| Wegener’s granulomatosis | M31.3 |  |  |  |
| Giant cell arteritis with polymyalgia rheumatica and other necrotizing vasculopathies | M31.5, M31.6 |  |  |  |
| Reiter’s disease | M02.3 |  |  |  |
| Rheumatoid arthritis | M05, M06 |  |  |  |
| Psoriatic arthropathies | M070, M072, M073 |  |  |  |
| Juvenile arthritis | M08 |  |  |  |
| Polyarteritis nodosa | M30.0 |  |  |  |
| Mucocutaneous lymph node syndrome (Kawasaki) | M30.3 |  |  |  |
| Hypersensitivity angiitis | M31.0 |  |  |  |
| Thrombotic microangiopathy | M31.1 |  |  |  |
| Wegener’s granulomatosis | M31.3 |  |  |  |
| Systemic lupus erythematosus | M32 |  |  |  |
| Dermatopolymyositis | M33 |  |  |  |
| Systemic sclerosis | M34 |  |  |  |
| Sicca syndrome (Sjögren) | M35.0 |  |  |  |
| Behcet’s disease | M35.2 | V139 |  |  |
| Polymyalgia rheumatica | M35.3 |  |  |  |
| Ankylosing spondylitis | M45 |  |  |  |
| Palmar fascial fibromatosis (Dupuytren) | M72.0 |  |  |  |

Abbreviations: ICD-10, International Classification of Diseases, 10^th^ Revision; RID, rare and incurable disease
