# Supplementary material for: Impact of metabolic dysfunction-associated steatotic liver disease on hepatocellular carcinoma risk in autoimmune hepatitis
Source: PLoS One. 2025 Jul 22;20(7):e0325066. doi: 10.1371/journal.pone.0325066 (PMC12282895; doi:10.1371/journal.pone.0325066)
Supplement: S2 Table — (DOCX) [file pone.0325066.s002.docx]

**Supporting information**

**S2 Table. Distribution of extrahepatic autoimmune diseases in autoimmune hepatitis patients (n=7,382) and matched cohort (n=58,538)**

|  | **Overall  (n=65,920)** | **Autoimmune hepatitis  (n=7,382)** | **Matched control (n=58,538)** | ***P* value** |
| --- | --- | --- | --- | --- |
| Extrahepatic autoimmune disease, n (%) | 6,924 (10.5) | 2,187 (29.6) | 4,737 (8.1) | <0.001 |
| Diseases of the blood and blood-forming organs | 144 (0.2) | 79 (1.1) | 65 (0.1) | <0.001 |
| Certain disorders involving the immune mechanism | 132 (0.2) | 116 (1.6) | 16 (0.0) | <0.001 |
| Endocrine disease | 1,440 (2.2) | 383 (5.2) | 1,057 (1.8) | <0.001 |
| Diseases of the nervous system | 67 (0.1) | 24 (0.3) | 43 (0.1) | <0.001 |
| Diseases of the eye | 431 (0.7) | 60 (0.8) | 371 (0.6) | 0.072 |
| Diseases of the circulatory system | 431 (0.7) | 60 (0.8) | 371 (0.6) | 0.072 |
| Diseases of the digestive system | 356 (0.5) | 137 (1.9) | 219 (0.4) | <0.001 |
| Diseases of the skin and subcutaneous tissue | 949 (1.4) | 252 (3.4) | 697 (1.2) | <0.001 |
| Diseases of the musculoskeletal system and connective tissue | 4,161 (6.3) | 1,562 (21.2) | 2,599 (4.4) | <0.001 |
