# Supplementary material for: Impact of metabolic dysfunction-associated steatotic liver disease on hepatocellular carcinoma risk in autoimmune hepatitis
Source: PLoS One. 2025 Jul 22;20(7):e0325066. doi: 10.1371/journal.pone.0325066 (PMC12282895; doi:10.1371/journal.pone.0325066)
Supplement: S3 Table — (DOCX) [file pone.0325066.s003.docx]

**Supporting information**

**S3 Table. Baseline characteristics of patients with autoimmune hepatitis (n=3805) stratified by the presence of MASLD in the two-year landmark analysis**

|  | **Overall** | **Without MASLD** | **With MASLD** | **P value** |
| --- | --- | --- | --- | --- |
|  | **(n=3,805)** | **(n=2,979)** | **(n=826)** |  |
| Age, years, mean (SD) | 57.5 (11.7) | 57.3 (11.9) | 58.3 (10.9) | 0.027 |
| Female sex, n (%) | 3,270 (85.9) | 2,635 (88.5) | 635 (76.9) | <0.001 |
| Diagnosis period, n (%) |  |  |  | 0.004 |
| 2007–2010 | 331 (8.7) | 276 (9.3) | 55 (6.7) |  |
| 2011–2015 | 1,852 (48.7) | 1,469 (49.3) | 383 (46.4) |  |
| 2016–2019 | 1,622 (42.6) | 1,234 (41.4) | 388 (47.0) |  |
| Socioeconomic status, n (%) |  |  |  | 0.098 |
| National health insurance | 3,614 (95.0) | 2,828 (95.0) | 786 (95.2) |  |
| Household income ≥70% | 1,591 (41.8) | 1,275 (42.8) | 316 (38.3) |  |
| Household income 30–70% | 1,221 (32.1) | 943 (31.7) | 278 (33.7) |  |
| Household income <30% | 802 (21.1) | 610 (20.5) | 192 (23.2) |  |
| Medical aid | 113 (3.0) | 86 (2.9) | 27 (3.3) |  |
| Unknown | 78 (2.0) | 65 (2.2) | 13 (1.6) |  |
| Comorbidities, n (%) |  |  |  |  |
| Hypertension | 1,554 (40.8) | 1,117 (37.5) | 437 (52.9) | <0.001 |
| Diabetes mellitus | 1,386 (36.4) | 991 (33.3) | 395 (47.8) | <0.001 |
| Dyslipidemia | 2,702 (71.0) | 2,051 (68.8) | 651 (78.8) | <0.001 |
| CCI score, mean (SD) | 3.2 (2.2) | 3.1 (2.1) | 3.4 (2.3) | <0.001 |
| Decompensated cirrhosis, n (%) | 210 (5.5) | 147 (4.9) | 63 (7.6) | 0.004 |
| Extrahepatic autoimmune disease, n (%) | 1,144 (30.1) | 932 (31.3) | 212 (25.7) | 0.002 |
| Medication use^*^, n (%) |  |  |  |  |
| Glucocorticoid | 1,808 (47.5) | 1,407 (47.2) | 401 (48.5) | 0.528 |
| Immunosuppressive agents^†^ | 1,480 (38.9) | 1,135 (38.1) | 345 (41.8) | 0.061 |
| Alcohol quantity (gram/week, n [%]) |  |  |  | 0.009 |
| <420 (for males) or <350 (for females) | 3,782 (99.4) | 2,956 (99.2) | 826 (100.0) |  |
| ≥420 (for males) or ≥350 (for females) or unknown | 23 (0.6) | 23 (0.8) | 0 (0.0) |  |
| Smoking, n (%) |  |  |  | 0.004 |
| Never | 3,288 (86.4) | 2,634 (88.4) | 654 (79.2) |  |
| Ever or unknown | 517 (13.6) | 11 (11.6) | 172 (20.8) |  |

Abbreviations: CCI, Charlson Comorbidity Index; MASLD, metabolic dysfunction-associated steatotic liver disease; SD, standard deviation.
^*^ Medication use was defined as the prescription of medications for more than 180 days within the two years following the diagnosis of autoimmune hepatitis.
^†^ Immunosuppressive agents included azathioprine, mercaptopurine, mycophenolate mofetil, tacrolimus, and cyclosporine, all of which were prescribed after the diagnosis of autoimmune hepatitis.
